# Supplementary figures and images for: Omics based approaches to decipher the leaf ionome and transcriptome changes in Solanum lycopersicum L. upon Tomato Brown Rugose Fruit Virus (ToBRFV) infection
Source: PLoS One. 2024 Nov 8;19(11):e0313335. doi: 10.1371/journal.pone.0313335 (PMC11548745; doi:10.1371/journal.pone.0313335)

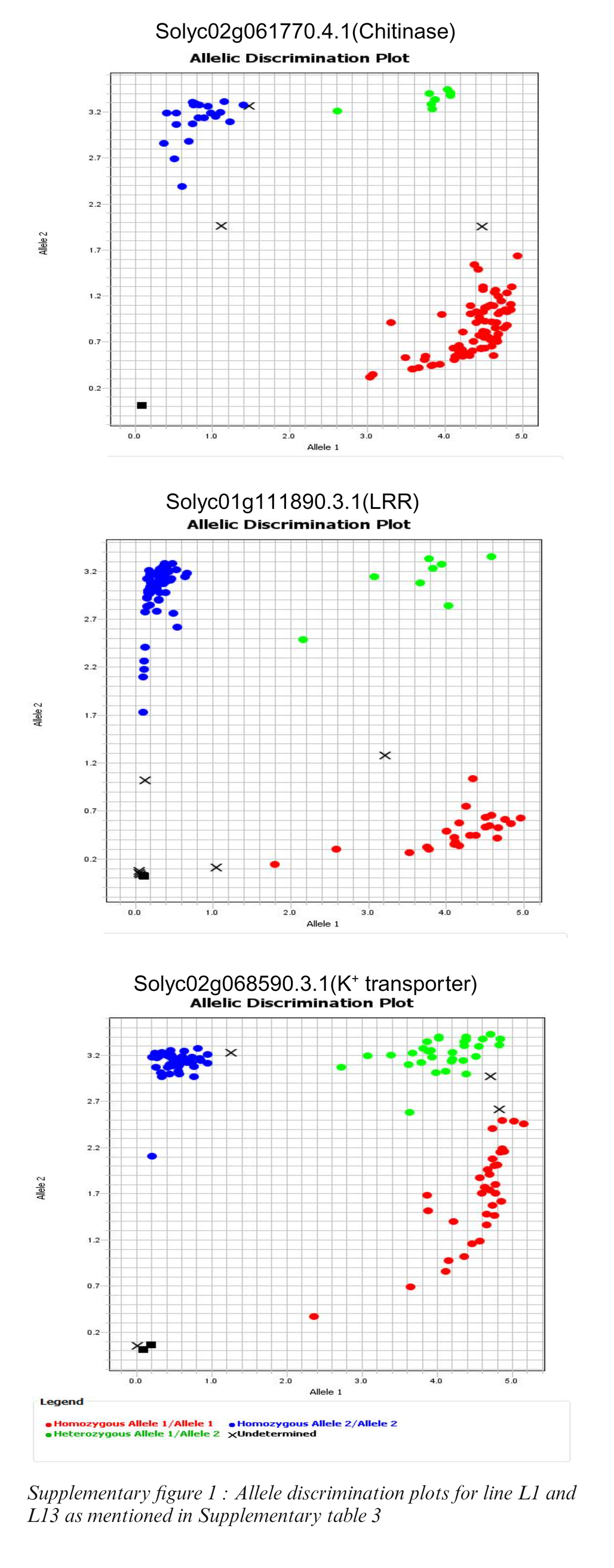

Supplement: S1 Fig — (TIF) [file pone.0313335.s001.tif]

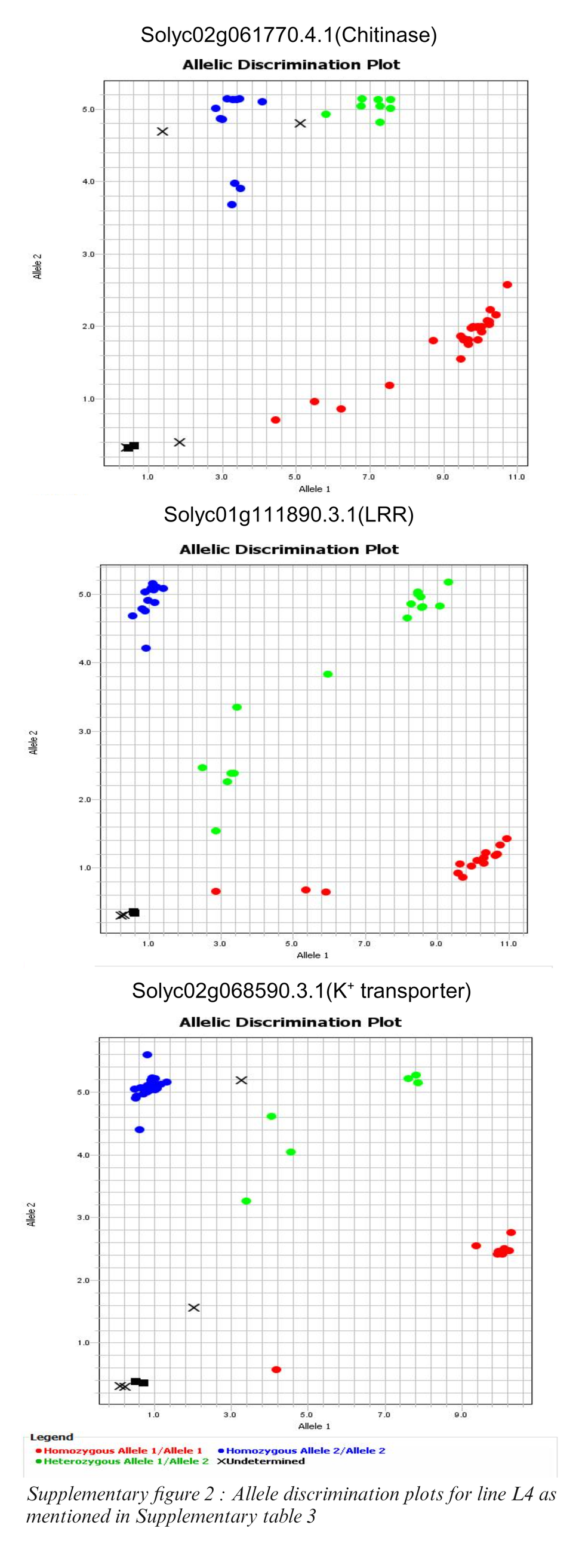

Supplement: S2 Fig — (TIF) [file pone.0313335.s002.tif]
