# Supplementary material for: Omics based approaches to decipher the leaf ionome and transcriptome changes in Solanum lycopersicum L. upon Tomato Brown Rugose Fruit Virus (ToBRFV) infection
Source: PLoS One. 2024 Nov 8;19(11):e0313335. doi: 10.1371/journal.pone.0313335 (PMC11548745; doi:10.1371/journal.pone.0313335)
Supplement: S1 Text — (DOCX) [file pone.0313335.s003.docx]

**>ch02_31290675 (Solyc02g061770.4.1 - Chitinase)**

GCATGAAGTCACAAAATATGTCAGGTAAGACAAAAGATGTTCAAAATTACAAAAAAAAATAAGTTCAAAAATAATGGGATCTTATTATAATTAGGAAAATGCCTCTGATATTTCGATTATAATCTAAAAATTATTTATACTTTATCCCAAAAGATAATATATGTAAATAAAGAATGAAATAAATAACATCTATGTAAAGCCCTTTTTGTTACAGTCATAATAATAATATTAATAATGTTATACAGCAACTACAATTATGGGGCAGCAGGGAGTGCAATAGGTGTGAATCTGCTAAACAACCCAGATTTAGTAGCCAATGATGCAGTAGTGTCATTCAAAACAGCGTTGTGGTTCTGGATGACAGCACAACAGCCAAAACCATCGGCACACGATGTCATTACTGGAAGATGGAGTCCCTCTGTTGCTGATTCTGCCGCTGGCCGCGTCCCGGGCTTTGGTGTAATCACTAACATCATCAATGGTGGAATGGAATGTAACAG[A/C]GGTTCAAATGCACTAATGGATAACAGAATTGGGTTCTATAGGAGATATTGTCAAATTTTGGGAGTTGATCCTGGTAATAATTTGGACTGTGCTAATCAAAGGCCATTTGGATAAAAGGCAACTTAATTAATTTTACTACAATAAGTTGCTGCTTTAATTTATATGTGTGATCCATATTTAGTACGTGACACATTAATAAGGTTTGGTCCATCTAAACTGTGTATTAGTTCGTTTGTGTTCTATAATGGAACTTCCATTTTGTCACGCAGGCGAAATAAAGTTAAAGTTTTATGCATTAATCAATTTTATCAAGAGTTCGTAGTCAAAAGGATAAATTTTTTCTTTAAAGAAAAACTAAAACGGCACATAAAAAAAAATTAACCAAAAGGGTAAAATGGATTCGTGGGAAGCGATTTTGTTCTGATAAAAATCCGTCGTTACAAAAAAATATAAATCGCTATCTTTGAAGCGATTTTCTAAAAAAAAAGAAACTTCTTTCT

**>ch01_90415583 (Solyc01g111890.3.1 - LRR)**

CCTGAGGAGCAAAATTGAAGATAACAAACAAGCTAATGACCAACTGAACAAATCATCAAATGAGTTTTCAGTTTTTCTTTCTTTTATTCTCATTATTCATTATATAAGCTCAAAGTAGACTCCATTTTATTTCACAATAAAGAGAAACCAAGAAAAACTCATACATGGATTTAGGAAGAGAAAATAAAAAAGCTAACCTGAGGTCTTTACAGTAGTGGCCGACCTGAGAGAGAAGTCTTCCACTGAAATCAGCGCAGTTATGAAGGCAAAGCTCTTGTAAATTCGGCTGAAGAAGACAATCAAGGGATGAATCATCAAGTCGCATGCAATCTACCTTCAAGCTACGAAGGTAAGGATTGGGAGGTAGCAACGGCCTTATATATTCAGCAGGTGGAGCAACATCCTTATAAGGAAACACAAAGTAAGAGAACCAGAATTGATGCTAAGTCATGTACAAGAACATTAGAAATGAAATAGACTTACAAGGAGATGAAAACTAG[A/G]AATGAAGCAAAGCATGTGTGAGGCACATGAATGAAAGGCCTTGCAAGTGCATGCCAGGGAACGAATGGAAGCTATGTCCAGCTTTGTCATCACAGTGGCCAATAGTGCTGATGGAAGAAGGTCCAGGCTCATGGGCTTGTGTGCGCCAGAATCTGAAGAAGACTTGGCCATTTTTCCTCTTTGCCAAACAGAACTCCGACAAGGCAACAAATTGAGTTAATGTGCAGATGAGATGTAAGTTGTGGGTAAGGGGTCGAAACAGGAAAATGTCAATTGCTCTAATCACTTAGTTGTTAGCAATGGAAAGAGGATATCAGATACTCTTTCTCCGGACAAACAAAAGCAACATTATTTGTTTCATTTGCTAGACGAAAATGCATAAAATCTACATAGAAATAATTATATATATTGTAACCTCTGCTTCTATCAAATTTCAAAAGTAGAAGAGTCCATTTCTCACCAAAATAACTTTACCTTTTCTGATAAATAGAAAATCATCC

**>Ch02_36559288 (Solyc02g068590.3.1 - K+ transporter)**

GTCAATCTAGTAGTAGCATAGAGACGGCTGACAATCAAAGTATTGTGGAGGAGCAGCTGCAGAAACTGGTGGATCAACGAATGAAACAGATCAAGTTGATTGCTGAAAAGGTTTCTTCGACAAAGGCAGCTGCTATGAAGGCAGAAGTAAAATTTCGACATGAAAAAAAGGAGTCTCTGTAAGGTTAAATTAGCTTAGTCTTTCAATTATATATGTCTGTGACTTGTTATAACTATTTGATATGAGGCAGCATTCTGCATTCTCTCTTGAACAAACTGTTATGTGTATCTTATAACAATTTGGTATGAGGCAGCATTGAACAAAACATTATGTGCAATCTTATATTGTTGCTATTGCTATTGTTAATGAAGTAGATTATCACTTCTGCTCAAGTTACAACTAGACATCTCATGATATAGATGGATAATATTCTTTGATATGATCAGTTCATTCATTTTGTTGAAACCAAAATTATACATAGAATATCTGTCCAACATTTA[A/C]TAGGCTCTCATGAGGGACATTGAAGATTACACTATTTTCCCTGCATATTTTCCTAAGAAACGCGTAAATATAGTCGATAGCAATTTTCTTGTAGAACCTAGAGTCCCTCCTTGCTCTAACTACAGTGTTTCCAAGAATGTGTACAACCCCAGTATCTCTACATCGATTTAAGAATTCCATTTCATCCGCCTCTGCCTGGCTGCTCTCGCGGCCTGATGATGTGACTGTGTTGTTGTGCCCTTGAGTAGGAGACTTTACAGGAATTATTGAGTCCACAGTCGAACATGTGTAGTCATTATTTCCTGTAGTTGAGTTGCCATTACTTCGCAGTAGGTAATCTGCTGAATGCTGAGTTTGCTGCCCGTATAAGCTGTATTCATCGGAGTCAGAGCAACCTTCCATCATATTCTCCAGCCGGACAAACAGGAAGAGGTTATCAAATAGCTTCCTCTCAAACTCCTCGTCTTTCTTATGGAGGTCCTTGTAACCATACCTCGCAA

*Supplementary Text 1: Sequences of the genes which were used to design rhAmp assay primer. Text highlighted in red and square brackets are the mono-allelic SNPs.*
