# Supplementary material for: Omics based approaches to decipher the leaf ionome and transcriptome changes in Solanum lycopersicum L. upon Tomato Brown Rugose Fruit Virus (ToBRFV) infection
Source: PLoS One. 2024 Nov 8;19(11):e0313335. doi: 10.1371/journal.pone.0313335 (PMC11548745; doi:10.1371/journal.pone.0313335)
Supplement: S3 Table — (DOCX) [file pone.0313335.s006.docx]

*Supplementary Table 3 : GenotypingResults_rhAmp*

| **Plate** | **Phenotype** | **202.1**  **Solyc02g061770.4.1**  **(Chitinase)** | **319.1**  **Solyc01g111890.3.1**  **(LRR)** | **565.1**  **Solyc02g068590.3.1**  **(K+ transporter)** |
| --- | --- | --- | --- | --- |
| L13 | S | Hom 2/2 | Hom 1/1 | Hom 1/1 |
| L13 | S | Hom 1/1 | Hom 1/1 | Hom 1/1 |
| L13 | S | Hom 1/1 | Het 1/2 | Hom 1/1 |
| L13 | S | Hom 2/2 | Hom 2/2 | Hom 2/2 |
| L13 | S | Hom 1/1 | Hom 2/2 | Undetermined |
| L13 | S | Hom 1/1 | Hom 2/2 | Undetermined |
| L13 | S | Hom 2/2 | Hom 2/2 | Hom 2/2 |
| L13 | S | Het 1/2 | Hom 2/2 | Hom 2/2 |
| L13 | S | Het 1/2 | Hom 2/2 | Het 1/2 |
| L13 | S | Hom 2/2 | Hom 2/2 | Hom 2/2 |
| L13 | S | Het 1/2 | Hom 2/2 | Hom 2/2 |
| L13 | S | Het 1/2 | Hom 2/2 | Het 1/2 |
| L1 | R | Hom 1/1 | Hom 2/2 | Het 1/2 |
| L1 | R | Hom 1/1 | Hom 2/2 | Hom 2/2 |
| L1 | R | Hom 1/1 | Hom 2/2 | Hom 2/2 |
| L1 | R | Hom 1/1 | Undetermined | Hom 1/1 |
| L1 | R | Hom 1/1 | Hom 2/2 | Hom 1/1 |
| L1 | R | Hom 1/1 | Undetermined | Het 1/2 |
| L1 | R | Hom 1/1 | Hom 2/2 | Hom 2/2 |
| L1 | R | Hom 1/1 | Undetermined | Hom 2/2 |
| L1 | R | Hom 1/1 | Hom 2/2 | Hom 2/2 |
| L1 | S | Hom 1/1 | Hom 2/2 | Het 1/2 |
| L1 | R | Hom 1/1 | Hom 2/2 | Hom 2/2 |
| L1 | R | Hom 1/1 | Hom 2/2 | Undetermined |
| L1 | R | Hom 1/1 | Hom 2/2 | Het 1/2 |
| L1 | R | Hom 1/1 | Hom 2/2 | Hom 1/1 |
| L1 | R | Hom 1/1 | Undetermined | Hom 2/2 |
| L1 | R | Hom 1/1 | Undetermined | Undetermined |
| L1 | R | Hom 1/1 | Hom 2/2 | Hom 1/1 |
| L1 | R | Hom 1/1 | Hom 2/2 | Het 1/2 |
| L1 | R | Hom 1/1 | Undetermined | Het 1/2 |
| L1 | R | Hom 1/1 | Undetermined | Hom 1/1 |
| L1 | R | Hom 1/1 | Undetermined | Het 1/2 |
| L1 | S | Hom 1/1 | Undetermined | Hom 1/1 |
| L1 | R | Hom 1/1 | Undetermined | Hom 1/1 |
| L1 | S | Hom 1/1 | Undetermined | Het 1/2 |
| L1 | R | Hom 1/1 | Undetermined | Het 1/2 |
| L1 | R | Hom 1/1 | Undetermined | Het 1/2 |
| L1 | S | Hom 1/1 | Undetermined | Het 1/2 |
| L1 | S | Hom 1/1 | Undetermined | Het 1/2 |
| L1 | S | Hom 1/1 | Hom 1/1 | Het 1/2 |
| L1 | R | Hom 1/1 | Hom 2/2 | Het 1/2 |
| L1 | S | Hom 1/1 | Hom 2/2 | Hom 1/1 |
| L1 | S | Hom 1/1 | Undetermined | Het 1/2 |
| L1 | R | Hom 1/1 | Hom 2/2 | Het 1/2 |
| L1 | R | Hom 1/1 | Hom 2/2 | Het 1/2 |
| L1 | S | Hom 1/1 | Hom 2/2 | Het 1/2 |
| L1 | R | Hom 1/1 | Hom 2/2 | Hom 2/2 |
| L1 | R | Hom 1/1 | Undetermined | Het 1/2 |
| L1 | R | Hom 1/1 | Hom 2/2 | Hom 2/2 |
| L1 | R | Hom 1/1 | Hom 2/2 | Het 1/2 |
| L1 | S | Hom 1/1 | Hom 2/2 | Het 1/2 |
| L1 | S | Hom 1/1 | Hom 2/2 | Hom 2/2 |
| L1 | R | Hom 1/1 | Hom 2/2 | Hom 1/1 |
| L1 | S | Hom 1/1 | Hom 2/2 | Het 1/2 |
| L1 | R | Hom 1/1 | Hom 1/1 | Undetermined |
| L1 | S | Hom 1/1 | Hom 2/2 | Hom 2/2 |
| L1 | S | Hom 2/2 | Hom 1/1 | Hom 2/2 |
| L1 | S | Hom 2/2 | Hom 1/1 | Hom 2/2 |
| L1 | S | Hom 2/2 | Hom 1/1 | Hom 2/2 |
| L1 | S | Hom 2/2 | Hom 1/1 | Hom 2/2 |
| L1 | S | Hom 1/1 | Hom 1/1 | Hom 1/1 |
| L1 | S | Hom 1/1 | Hom 1/1 | Hom 1/1 |
| L1 | S | Hom 1/1 | Hom 1/1 | Hom 1/1 |
| L1 | S | Hom 1/1 | Hom 1/1 | Hom 1/1 |
| L1 | S | Hom 1/1 | Hom 1/1 | Hom 1/1 |
| L1 | S | Hom 1/1 | Hom 1/1 | Hom 2/2 |
| L1 | S | Hom 1/1 | Hom 1/1 | Hom 2/2 |
| L1 | S | Hom 1/1 | Hom 1/1 | Hom 2/2 |
| L1 | R | Hom 1/1 | Hom 1/1 | Hom 2/2 |
| L1 | S | Hom 1/1 | Undetermined | Undetermined |
| L1 | S | Hom 1/1 | Undetermined | Het 1/2 |
| L1 | S | Hom 1/1 | Het 1/2 | Hom 1/1 |
| L1 | S | Hom 1/1 | Het 1/2 | Hom 2/2 |
| L1 | S | Hom 1/1 | Hom 2/2 | Hom 2/2 |
| L1 | R | Hom 1/1 | Hom 2/2 | Hom 2/2 |
| L1 | S | Hom 2/2 | Hom 2/2 | Hom 2/2 |
| L1 | S | Hom 2/2 | Hom 2/2 | Hom 2/2 |
| L1 | S | Hom 2/2 | Hom 2/2 | Hom 2/2 |
| L1 | S | Hom 2/2 | Hom 2/2 | Hom 2/2 |
| L1 | R | Hom 1/1 | Hom 1/1 | Hom 2/2 |
| L1 | S | Hom 1/1 | Hom 1/1 | Hom 2/2 |
| L1 | R | Hom 1/1 | Hom 1/1 | Hom 2/2 |
| L1 | S | Hom 1/1 | Hom 1/1 | Hom 2/2 |
| L1 | S | Hom 2/2 | Hom 2/2 | Hom 1/1 |
| L1 | S | Hom 2/2 | Hom 2/2 | Hom 1/1 |
| L1 | S | Hom 2/2 | Hom 2/2 | Hom 1/1 |
| L1 | S | Hom 2/2 | Hom 2/2 | Hom 1/1 |
| L1 | S | Hom 1/1 | Hom 2/2 | Hom 1/1 |
| L1 | R | Hom 2/2 | Hom 2/2 | Hom 2/2 |
| L1 | R | Hom 2/2 | Hom 2/2 | Hom 2/2 |
| L2 | S | Undetermined | Hom 1/1 | Hom 1/1 |
| L2 | S | Undetermined | Hom 1/1 | Hom 1/1 |
| L2 | S | Undetermined | Hom 1/1 | Hom 1/1 |
| L2 | S | Undetermined | Hom 1/1 | Hom 1/1 |
| L2 | S | Undetermined | Hom 1/1 | Hom 1/1 |
| L2 | S | Undetermined | Hom 1/1 | Hom 1/1 |
| L2 | S | Undetermined | Hom 1/1 | Hom 1/1 |
| L2 | S | Undetermined | Hom 1/1 | Hom 1/1 |
| L2 | S | Undetermined | Hom 1/1 | Hom 1/1 |
| L2 | S | Undetermined | Hom 1/1 | Hom 1/1 |
| L2 | S | Undetermined | Hom 1/1 | Hom 1/1 |
| L2 | S | Undetermined | Hom 1/1 | Hom 1/1 |
| L2 | S | Undetermined | Hom 1/1 | Hom 1/1 |
| L2 | S | Undetermined | Hom 1/1 | Hom 1/1 |
| L2 | S | Undetermined | Hom 1/1 | Hom 1/1 |
| L2 | S | Undetermined | Hom 1/1 | Hom 1/1 |
| L2 | S | Undetermined | Hom 1/1 | Hom 1/1 |
| L2 | S | Undetermined | Hom 1/1 | Hom 1/1 |
| L2 | S | Undetermined | Hom 1/1 | Hom 1/1 |
| L2 | S | Undetermined | Hom 1/1 | Hom 1/1 |
| L2 | S | Undetermined | Hom 1/1 | Hom 1/1 |
| L2 | S | Undetermined | Hom 1/1 | Hom 1/1 |
| L2 | S | Undetermined | Hom 1/1 | Hom 1/1 |
| L2 | S | Undetermined | Hom 1/1 | Hom 1/1 |
| L2 | S | Undetermined | Hom 1/1 | Hom 1/1 |
| L2 | S | Undetermined | Hom 1/1 | Hom 1/1 |
| L2 | S | Undetermined | Hom 1/1 | Hom 1/1 |
| L2 | S | Undetermined | Hom 1/1 | Hom 1/1 |
| L2 | S | Undetermined | Hom 1/1 | Hom 1/1 |
| L2 | S | Undetermined | Hom 1/1 | Hom 2/2 |
| L2 | S | Undetermined | Hom 1/1 | Hom 2/2 |
| L2 | S | Undetermined | Hom 1/1 | Hom 2/2 |
| L2 | R | Undetermined | Hom 2/2 | Hom 2/2 |
| L2 | R | Undetermined | Hom 2/2 | Hom 2/2 |
| L2 | R | Undetermined | Undetermined | Hom 2/2 |
| L2 | R | Undetermined | Hom 2/2 | Hom 2/2 |
| L14 | R | Hom 1/1 | Het 1/2 | Het 1/2 |
| L14 | R | Hom 1/1 | Het 1/2 | Het 1/2 |
| L14 | R | Het 1/2 | Undetermined | Hom 1/1 |
| L14 | R | Undetermined | Undetermined | Hom 1/1 |
| L14 | S | Hom 1/1 | Hom 1/1 | Hom 2/2 |
| L14 | S | Hom 1/1 | Hom 1/1 | Hom 2/2 |
| L14 | S | Hom 1/1 | Hom 1/1 | Hom 2/2 |
| L14 | S | Hom 1/1 | Hom 1/1 | Hom 2/2 |
| L12 | R | Hom 1/1 | Hom 2/2 | Hom 2/2 |
| L12 | R | Hom 1/1 | Hom 2/2 | Hom 2/2 |
| L12 | R | Hom 1/1 | Hom 2/2 | Hom 2/2 |
| L12 | R | Hom 1/1 | Hom 2/2 | Hom 2/2 |
| L12 | R | Hom 1/1 | Hom 2/2 | Het 1/2 |
| L12 | R | Hom 1/1 | Hom 2/2 | Het 1/2 |
| L15 | R | Het 1/2 | Undetermined | Hom 1/1 |
| L15 | R | Het 1/2 | Het 1/2 | Het 1/2 |
| L15 | R | Het 1/2 | Undetermined | Hom 1/1 |
| L15 | R | Het 1/2 | Undetermined | Hom 1/1 |
| L15 | R | Undetermined | Undetermined | Het 1/2 |
| L3 | R | Het 1/2 | Hom 2/2 | Het 1/2 |
| L3 | R | Het 1/2 | Hom 2/2 | Het 1/2 |
| L3 | R | Het 1/2 | Hom 2/2 | Het 1/2 |
| L3 | R | Het 1/2 | Hom 2/2 | Het 1/2 |
| L3 | R | Het 1/2 | Het 1/2 | Het 1/2 |
| L3 | R | Het 1/2 | Het 1/2 | Het 1/2 |
| L3 | R | Het 1/2 | Het 1/2 | Het 1/2 |
| L3 | R | Het 1/2 | Het 1/2 | Het 1/2 |
| L4 | S | Undetermined | Het 1/2 | Hom 2/2 |
| L4 | S | Hom 2/2 | Het 1/2 | Hom 2/2 |
| L4 | S | Hom 1/1 | Hom 2/2 | Undetermined |
| L4 | S | Hom 1/1 | Hom 2/2 | Hom 2/2 |
| L4 | S | Het 1/2 | Het 1/2 | Hom 2/2 |
| L4 | S | Het 1/2 | Het 1/2 | Hom 2/2 |
| L4 | S | Het 1/2 | Het 1/2 | Het 1/2 |
| L4 | S | Het 1/2 | Het 1/2 | Het 1/2 |
| L4 | R | Het 1/2 | Hom 2/2 | Hom 2/2 |
| L4 | R | Het 1/2 | Hom 2/2 | Het 1/2 |
| L4 | S | Hom 1/1 | Hom 2/2 | Hom 1/1 |
| L4 | S | Hom 1/1 | Hom 2/2 | Hom 1/1 |
| L4 | S | Hom 1/1 | Hom 2/2 | Hom 1/1 |
| L4 | S | Hom 1/1 | Hom 2/2 | Hom 1/1 |
| L4 | R | Hom 1/1 | Hom 1/1 | Hom 2/2 |
| L4 | R | Hom 1/1 | Hom 1/1 | Hom 2/2 |
| L4 | S | Hom 2/2 | Het 1/2 | Hom 2/2 |
| L4 | S | Hom 2/2 | Het 1/2 | Hom 2/2 |
| L4 | R | Hom 2/2 | Hom 1/1 | Hom 2/2 |
| L4 | R | Hom 2/2 | Hom 1/1 | Hom 2/2 |
| L4 | R | Hom 1/1 | Hom 2/2 | Hom 2/2 |
| L4 | R | Hom 1/1 | Hom 2/2 | Hom 2/2 |
| L4 | S | Hom 1/1 | Hom 1/1 | Hom 2/2 |
| L4 | S | Hom 1/1 | Hom 1/1 | Hom 2/2 |
| L4 | S | Hom 2/2 | Het 1/2 | Hom 2/2 |
| L4 | S | Hom 1/1 | Hom 1/1 | Hom 1/1 |
| L4 | S | Hom 1/1 | Hom 2/2 | Hom 2/2 |
| L4 | S | Het 1/2 | Het 1/2 | Hom 2/2 |
| L4 | S | Het 1/2 | Het 1/2 | Het 1/2 |
| L4 | R | Hom 1/1 | Hom 1/1 | Hom 2/2 |
| L4 | R | Het 1/2 | Hom 2/2 | Hom 2/2 |
| L4 | S | Hom 1/1 | Undetermined | Hom 1/1 |
| L4 | S | Hom 1/1 | Hom 2/2 | Hom 1/1 |
| L4 | S | Hom 1/1 | Hom 2/2 | Het 1/2 |
| L4 | R | Hom 1/1 | Hom 1/1 | Hom 2/2 |
| L4 | R | Hom 1/1 | Hom 1/1 | Hom 2/2 |
| L4 | S | Hom 2/2 | Het 1/2 | Hom 2/2 |
| L4 | S | Hom 1/1 | Hom 1/1 | Hom 1/1 |
| L4 | R | Hom 2/2 | Hom 1/1 | Hom 2/2 |
| L4 | S | Hom 1/1 | Het 1/2 | Hom 2/2 |
| L4 | R | Hom 1/1 | Hom 2/2 | Hom 2/2 |
| L4 | S | Hom 1/1 | Hom 1/1 | Hom 2/2 |
| L4 | R | Hom 1/1 | Hom 1/1 | Hom 2/2 |
| L5 | R | Hom 1/1 | Hom 1/1 | Hom 1/1 |
| L5 | R | Hom 1/1 | Hom 1/1 | Hom 1/1 |
| L5 | R | Hom 1/1 | Hom 1/1 | Hom 1/1 |
| L6 | S | Hom 1/1 | Hom 1/1 | Hom 1/1 |
| L6 | S | Hom 1/1 | Hom 1/1 | Hom 1/1 |
| L6 | S | Hom 1/1 | Hom 1/1 | Hom 1/1 |
| L5 | R | Hom 1/1 | Hom 1/1 | Hom 1/1 |
| L6 | S | Hom 1/1 | Hom 1/1 | Hom 1/1 |
| L7 | R | Undetermined | Het 1/2 | Het 1/2 |
| L7 | R | Het 1/2 | Het 1/2 | Het 1/2 |
| L7 | R | Het 1/2 | Het 1/2 | Het 1/2 |
| L7 | R | Het 1/2 | Het 1/2 | Het 1/2 |
| L7 | R | Het 1/2 | Het 1/2 | Het 1/2 |
| L8 | R | Hom 1/1 | Het 1/2 | Hom 1/1 |
| L8 | R | Hom 1/1 | Het 1/2 | Hom 1/1 |
| L8 | R | Hom 1/1 | Het 1/2 | Hom 1/1 |
| L8 | R | Hom 1/1 | Het 1/2 | Hom 1/1 |
| L8 | R | Hom 1/1 | Het 1/2 | Hom 1/1 |
| L9 | S | Hom 2/2 | Hom 2/2 | Undetermined |
| L9 | S | Hom 2/2 | Hom 2/2 | Undetermined |
| L9 | S | Hom 2/2 | Hom 2/2 | Undetermined |
| L9 | R | Hom 1/1 | Hom 1/1 | Undetermined |
| L9 | R | Hom 1/1 | Hom 1/1 | Undetermined |
| L9 | R | Hom 1/1 | Hom 1/1 | Undetermined |
| L9 | R | Hom 1/1 | Hom 1/1 | Undetermined |
| L9 | R | Hom 1/1 | Hom 1/1 | Undetermined |
| L9 | R | Hom 1/1 | Hom 1/1 | Undetermined |
| L9 | R | Hom 1/1 | Hom 1/1 | Undetermined |
| L9 | R | Hom 1/1 | Hom 1/1 | Undetermined |
| L9 | R | Hom 1/1 | Hom 1/1 | Undetermined |
| L9 | R | Hom 1/1 | Hom 1/1 | Undetermined |
| L9 | R | Hom 1/1 | Hom 1/1 | Undetermined |
| L9 | R | Hom 2/2 | Hom 1/1 | Undetermined |
| L9 | R | Hom 2/2 | Hom 1/1 | Undetermined |
| L9 | R | Hom 2/2 | Hom 1/1 | Undetermined |
| L9 | R | Hom 2/2 | Hom 1/1 | Undetermined |
| L9 | S | Hom 2/2 | Hom 1/1 | Undetermined |
| L9 | S | Hom 2/2 | Hom 1/1 | Undetermined |
| L9 | S | Hom 2/2 | Hom 1/1 | Undetermined |
| L9 | S | Hom 1/1 | Hom 1/1 | Undetermined |
| L9 | S | Hom 1/1 | Hom 1/1 | Undetermined |
| L9 | S | Hom 1/1 | Hom 1/1 | Undetermined |
| L9 | S | Hom 1/1 | Hom 1/1 | Undetermined |
| L9 | S | Hom 1/1 | Hom 2/2 | Undetermined |
| L9 | S | Hom 1/1 | Hom 2/2 | Undetermined |
| L9 | S | Hom 1/1 | Hom 2/2 | Undetermined |
| L9 | S | Hom 1/1 | Hom 2/2 | Undetermined |
| L9 | S | Hom 1/1 | Hom 2/2 | Undetermined |
| L9 | S | Hom 1/1 | Hom 2/2 | Undetermined |
| L9 | S | Hom 1/1 | Hom 2/2 | Undetermined |
| L9 | S | Hom 1/1 | Hom 2/2 | Undetermined |
| L9 | S | Hom 1/1 | Hom 2/2 | Undetermined |
| L9 | R | Hom 1/1 | Hom 2/2 | Undetermined |
| L9 | R | Hom 1/1 | Hom 2/2 | Undetermined |
| L9 | R | Hom 1/1 | Hom 2/2 | Undetermined |
| L9 | R | Hom 1/1 | Hom 2/2 | Undetermined |
| L9 | R | Hom 1/1 | Hom 2/2 | Undetermined |
| L9 | R | Hom 1/1 | Hom 2/2 | Undetermined |
| L9 | R | Hom 1/1 | Hom 2/2 | Undetermined |
| L9 | R | Hom 1/1 | Hom 2/2 | Undetermined |
| L9 | R | Hom 1/1 | Hom 2/2 | Undetermined |
| L9 | R | Hom 1/1 | Hom 2/2 | Undetermined |
| L9 | R | Hom 2/2 | Hom 2/2 | Undetermined |
| L9 | R | Hom 2/2 | Hom 2/2 | Undetermined |
| L9 | R | Hom 2/2 | Hom 2/2 | Undetermined |
| L9 | S | Hom 2/2 | Hom 2/2 | Undetermined |
| L9 | R | Hom 2/2 | Hom 2/2 | Undetermined |
| L9 | S | Hom 2/2 | Hom 2/2 | Undetermined |
| L9 | R | Het 1/2 | Hom 2/2 | Undetermined |
| L9 | R | Het 1/2 | Hom 2/2 | Undetermined |
| L9 | S | Het 1/2 | Hom 2/2 | Undetermined |
| L9 | R | Het 1/2 | Hom 2/2 | Undetermined |
| L9 | R | Hom 2/2 | Hom 2/2 | Undetermined |
| L9 | S | Hom 2/2 | Hom 2/2 | Undetermined |
| L9 | S | Hom 2/2 | Hom 2/2 | Undetermined |
| L9 | R | Hom 2/2 | Hom 2/2 | Undetermined |
| L9 | S | Hom 2/2 | Hom 2/2 | Undetermined |
| L9 | R | Hom 2/2 | Hom 2/2 | Undetermined |
| L9 | R | Hom 1/1 | Hom 2/2 | Undetermined |
| L9 | R | Hom 1/1 | Hom 2/2 | Undetermined |
| L9 | S | Hom 1/1 | Hom 2/2 | Undetermined |
| L9 | S | Hom 1/1 | Hom 2/2 | Undetermined |
| L9 | S | Hom 2/2 | Hom 2/2 | Undetermined |
| L9 | S | Hom 2/2 | Hom 2/2 | Undetermined |
| L9 | S | Hom 2/2 | Hom 2/2 | Undetermined |
| L9 | S | Hom 2/2 | Hom 2/2 | Undetermined |
| L9 | S | Hom 2/2 | Hom 2/2 | Undetermined |
| L9 | S | Het 1/2 | Hom 2/2 | Undetermined |
| L9 | S | Hom 2/2 | Hom 2/2 | Undetermined |
| L9 | R | Het 1/2 | Hom 2/2 | Undetermined |
| L9 | R | Hom 1/1 | Hom 2/2 | Undetermined |
| L9 | R | Hom 2/2 | Hom 2/2 | Undetermined |
| L9 | R | Hom 2/2 | Hom 2/2 | Undetermined |
| L9 | R | Hom 2/2 | Hom 2/2 | Undetermined |
| L9 | S | Hom 2/2 | Hom 2/2 | Undetermined |
| L9 | S | Hom 2/2 | Hom 2/2 | Undetermined |
| L9 | S | Hom 2/2 | Hom 2/2 | Undetermined |
| L9 | S | Hom 2/2 | Hom 2/2 | Undetermined |
| L9 | S | Het 1/2 | Hom 2/2 | Undetermined |
| L9 | R | Het 1/2 | Hom 2/2 | Undetermined |
| L9 | S | Hom 2/2 | Hom 2/2 | Undetermined |
| L9 | S | Hom 2/2 | Hom 2/2 | Undetermined |
| L9 | S | Hom 1/1 | Hom 2/2 | Undetermined |
| L9 | R | Hom 2/2 | Hom 2/2 | Undetermined |
| L9 | R | Het 1/2 | Hom 2/2 | Undetermined |
| L9 | S | Hom 2/2 | Hom 2/2 | Undetermined |
| L9 | R | Hom 1/1 | Hom 2/2 | Undetermined |
| L10 | R | Hom 1/1 | Hom 2/2 | Undetermined |
| L10 | R | Hom 1/1 | Hom 2/2 | Undetermined |
| L10 | R | Hom 1/1 | Hom 2/2 | Undetermined |
| L10 | R | Hom 1/1 | Hom 2/2 | Undetermined |
| L10 | R | Hom 1/1 | Hom 2/2 | Undetermined |
| L10 | R | Hom 1/1 | Hom 2/2 | Undetermined |
| L10 | R | Hom 1/1 | Hom 2/2 | Undetermined |
| L10 | R | Hom 1/1 | Hom 2/2 | Undetermined |
| L10 | R | Hom 1/1 | Hom 2/2 | Undetermined |
| L10 | R | Hom 1/1 | Hom 2/2 | Undetermined |
| L10 | R | Hom 1/1 | Hom 2/2 | Undetermined |
| L10 | R | Hom 1/1 | Hom 2/2 | Undetermined |
| L10 | R | Hom 1/1 | Hom 2/2 | Undetermined |
| L10 | R | Hom 1/1 | Hom 2/2 | Undetermined |
| L10 | R | Hom 1/1 | Hom 2/2 | Undetermined |
| L10 | R | Hom 1/1 | Hom 2/2 | Undetermined |
| L10 | R | Hom 1/1 | Hom 2/2 | Undetermined |
| L10 | R | Hom 1/1 | Hom 2/2 | Undetermined |
| L10 | R | Hom 1/1 | Hom 2/2 | Undetermined |
| L10 | R | Hom 1/1 | Hom 2/2 | Undetermined |
| L10 | R | Het 1/2 | Hom 2/2 | Undetermined |
| L10 | R | Hom 1/1 | Hom 2/2 | Undetermined |
| L10 | R | Hom 1/1 | Hom 2/2 | Undetermined |
| L10 | R | Hom 1/1 | Hom 2/2 | Undetermined |
| L10 | R | Het 1/2 | Hom 2/2 | Undetermined |
| L10 | S | Het 1/2 | Hom 2/2 | Undetermined |
| L10 | R | Hom 1/1 | Hom 2/2 | Undetermined |
| L10 | R | Het 1/2 | Hom 2/2 | Undetermined |
| L10 | R | Hom 1/1 | Hom 2/2 | Undetermined |
| L10 | S | Het 1/2 | Hom 2/2 | Undetermined |
| L10 | S | Het 1/2 | Hom 2/2 | Undetermined |
| L10 | R | Hom 2/2 | Het 1/2 | Undetermined |
| L10 | R | Het 1/2 | Het 1/2 | Undetermined |
| L10 | R | Hom 1/1 | Hom 1/1 | Undetermined |
| L10 | R | Hom 1/1 | Het 1/2 | Undetermined |
| L10 | R | Het 1/2 | Hom 1/1 | Undetermined |
| L10 | R | Hom 1/1 | Het 1/2 | Undetermined |
| L10 | R | Het 1/2 | Hom 2/2 | Undetermined |
| L10 | R | Hom 2/2 | Hom 1/1 | Undetermined |
| L10 | R | Hom 2/2 | Hom 1/1 | Undetermined |
| L10 | R | Hom 2/2 | Hom 1/1 | Undetermined |
| L10 | R | Hom 2/2 | Hom 1/1 | Undetermined |
| L10 | R | Hom 2/2 | Hom 1/1 | Undetermined |
| L10 | R | Hom 2/2 | Hom 1/1 | Undetermined |
| L10 | R | Hom 2/2 | Hom 1/1 | Undetermined |
| L10 | R | Hom 1/1 | Hom 2/2 | Undetermined |
| L10 | R | Het 1/2 | Het 1/2 | Undetermined |
| L10 | R | Hom 1/1 | Het 1/2 | Undetermined |
| L10 | R | Hom 2/2 | Hom 2/2 | Undetermined |
| L10 | S | Het 1/2 | Hom 1/1 | Undetermined |
| L10 | R | Het 1/2 | Hom 2/2 | Undetermined |
| L10 | R | Hom 1/1 | Hom 2/2 | Undetermined |
| L10 | R | Hom 1/1 | Het 1/2 | Undetermined |
| L10 | R | Hom 1/1 | Het 1/2 | Undetermined |
| L10 | R | Hom 1/1 | Het 1/2 | Undetermined |
| L10 | R | Hom 1/1 | Het 1/2 | Undetermined |
| L10 | R | Hom 1/1 | Het 1/2 | Undetermined |
| L10 | R | Het 1/2 | Hom 1/1 | Undetermined |
| L10 | R | Het 1/2 | Hom 1/1 | Undetermined |
| L10 | R | Hom 2/2 | Het 1/2 | Undetermined |
| L10 | R | Hom 1/1 | Het 1/2 | Undetermined |
| L10 | R | Het 1/2 | Hom 2/2 | Undetermined |
| L10 | R | Het 1/2 | Hom 1/1 | Undetermined |
| L11 | R | Het 1/2 | Undetermined | Hom 2/2 |
| L11 | R | Het 1/2 | Hom 2/2 | Hom 2/2 |
| L11 | R | Het 1/2 | Het 1/2 | Undetermined |
| L11 | R | Het 1/2 | Het 1/2 | Hom 2/2 |
| L11 | R | Undetermined | Het 1/2 | Hom 2/2 |
| L11 | R | Het 1/2 | Het 1/2 | Hom 2/2 |
| L11 | R | Hom 2/2 | Het 1/2 | Hom 2/2 |
| L11 | R | Hom 2/2 | Het 1/2 | Hom 2/2 |
| L11 | R | Undetermined | Het 1/2 | Hom 2/2 |
| L11 | R | Undetermined | Het 1/2 | Hom 2/2 |
| L11 | R | Hom 2/2 | Het 1/2 | Undetermined |
| L11 | R | Het 1/2 | Het 1/2 | Hom 2/2 |
| L11 | R | Het 1/2 | Het 1/2 | Hom 2/2 |
| L11 | R | Het 1/2 | Het 1/2 | Hom 2/2 |
| L11 | R | Het 1/2 | Het 1/2 | Hom 2/2 |

| ***Notes :*** |  |
| --- | --- |
| **Solyc02g061770.4.1 (Chitinase)**  Allele 1: T  Allele 2: G | **Solyc02g068590.3.1 (K+ transporter)**  Allele 1: A  Allele 2: C |
| **Solyc01g111890.3.1 (LRR)**  Allele 1: T  Allele 2: C |  |
